# Retinal Lineage Therapeutic Specific Effect of Human Orbital and Abdominal Adipose-Derived Mesenchymal Stem Cells

**DOI:** 10.1155/2021/7022247

**Published:** 2021-10-19

**Authors:** Bryan Krief, Shira Weisthal Algor, Itay Nakdimon, Ayala Elhikis, Moshe Benhamou, Anouk Savir Kadmon, Shay Keren, Oded Ohana, Ilan Feldman, Ran Ben Cnaan, Igal Leibovitch, Anat Loewenstein, Adiel Barak, Aya Barzelay

**Affiliations:** ^1^Department of Ophthalmology, Tel-Aviv Sourasky Medical Center, Tel-Aviv 6423906, Israel; ^2^Sackler Faculty of Medicine, Tel-Aviv University, Tel-Aviv 6997801, Israel

## Abstract

Retinal degenerative diseases are one of the main causes of complete blindness in aged population. In this study, we compared the therapeutic potential for retinal degeneration of human mesenchymal stem cells derived from abdominal subcutaneous fat (ABASCs) or from orbital fat (OASCs) due to their accessibility and mutual embryonic origin with retinal tissue, respectively. OASCs were found to protect RPE cells from cell death and were demonstrated to increase early RPE precursor markers, while ABASCs showed a raise in retinal precursor marker expression. Subretinal transplantation of OASCs in a mouse model of retinal degeneration led to restoration of the RPE layer while transplantation of ABASCs resulted in a significant restoration of the photoreceptor layer. Taken together, we demonstrated a lineage-specific therapeutic effect for either OASCs or ABASCs in retinal regeneration.

## 1. Introduction

Blindness and vision impairment affect more than 2 billion people worldwide and will inevitably continue to increase due to population age. Oxidative stress, inflammation, and degeneration of retinal cells are notably implicated in the development of several eye pathologies, in particular, in age-related macular degeneration (AMD) [1, 2].

The retinal pigment epithelium (RPE) is crucial for the retinal function, as it participates in nutrition, protection against photo/light oxidation, secretion of proteins required for retinal homeostasis [3], immune privilege of the eye [4], and phagocytosis of photoreceptor disk membranes [5]. RPE cells and photoreceptors are both specifically sensitive to oxidative damage that leads to their apoptosis [2]. Degeneration of RPE is one of the main pathologic factors of AMD [6]. RPE is not directly involved in the vision process; although, it allows the survival of photoreceptors [7] mainly implicated in the visual phototransduction whose mechanism was described by George Wald (Nobel Price 1967). One of the most extensively studied treatment option for RPE degeneration is RPE transplantation that may prevent photoreceptor cell death. Recently, stem cell therapy has been proposed as a possible option for cell replacement therapy in AMD.

Mesenchymal stem cells (MSCs) are especially attractive population for cell therapy purposes, besides the fact that they can serve as an autologous cell source or even allogenic cell source [8], they are also multipotent, and their paracrine activity promotes cell survival and antioxidative properties [9, 10]. MSCs are able to differentiate into osteocytes, chondrocytes, adipocytes, and to cells of an entirely distinct lineage, including neuron-like cells [10, 11]. The paracrine activation properties of MSCs are known to modulate the microenvironment of the diseased tissues by secretion of cytokines that protect injured cells, promote survival, and activate endogenous repair mechanisms [10, 12] including by an antioxidant effect [13]. MSCs can be isolated from different tissues as bone marrow and adipose tissue. A special advantage is given to adipose-derived MSCs (ASCs) since the harvesting of adipose tissue may be conducted in a minimal invasive procedure as we have recently shown [14] and could be isolated from many sites [15, 16]. We have recently demonstrated the beneficial effect of MSCs harvested from subcutaneous fat, in protection of RPE from oxidative stress-induced cell death in vitro, as well as their therapeutic effect with the salvage of both RPE and photoreceptor layers in an in vivo model of retinal degeneration after a week of treatment [10]. Most adipose tissues, including subcutaneous, are derived from mesoderm. Interestingly, the orbital fat derives from neural crest origin [17], like most ocular and orbital tissues origin [18], and specifically shares a mutual embryonic origin with RPE [19]. MSCs are known to be responsive to the niche they reside in [20], and it was shown for instance that ASC derived from pericardial fat is more potent in therapy for ischemic cardiomyocytes than ASCs harvested from abdominal or subcutaneous adipose tissue [20]. Hence, orbital fat-derived MSCs are a unique population with great interest when considering a potential for retinal repair. Thus, in this study, we examined whether human orbital adipose-derived mesenchymal stem cells have therapeutic advantage in retinal regeneration, when compared to human abdominal mesenchymal stem cells harvested from subcutaneous fat (ABASCs).

To this end, we studied the phenotypic characterization of OASCs, and we evaluated their protective effect on RPE cell survival when exposed to oxidative stress and their cytokine secretion profile when compared to ABASCs. Next, we studied the ability of OASCs to differentiate toward RPE compared to ABASCs. Finally, we investigated the long-term effect of subretinal transplantation of ABASCs and OASCs in a mouse model of retinal degeneration.

## 2. Results

### 2.1. Characterization and Multipotency of OASCs

**Figure 1:**
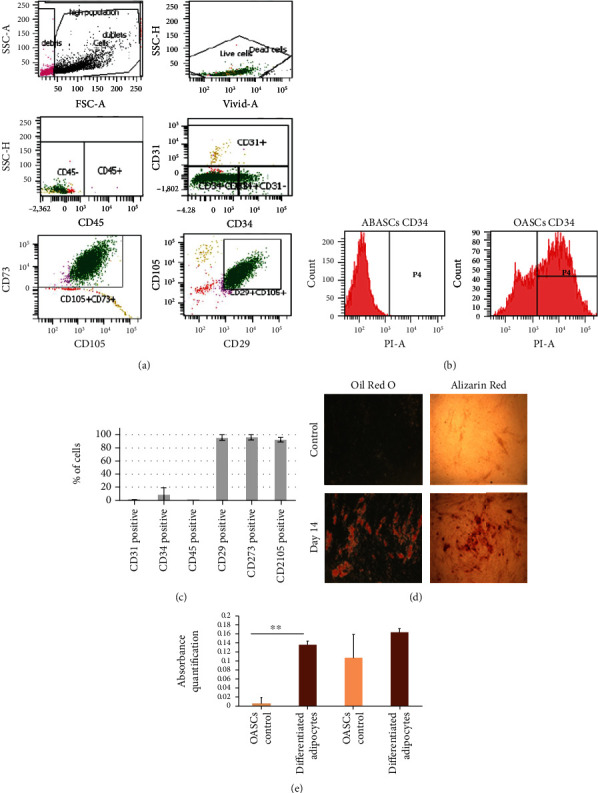
Characterization of OASCs by surface phenotype and differentiation potential. (a)–(c) OASCs were immunostained for CD29, CD31, CD34, CD45, CD73, and CD105 and analyzed by FACS. OASCs exhibited classic MSC phenotype when compared to ABASCs. (b, c) CD34 expression in OASCs was evaluated and compared to that observed in ABASCs. (d, e) Multipotency of OASCs shown by staining for Oil Red O and Alizarin Red and markers for adipocytes and osteocytes. Data are represented as mean ± SD.

OASCs positively expressed classic MSC markers (CD29: 93.5 ± 6.9%; CD73: 93.9 ± 7.6%; CD105: 90.9 ± 5.5%) and were negative for endothelial and hematopoietic markers (CD31: 1.3 ± 1.7%; CD45: 0.1 ± 0.1%), as well as varying levels of CD34, which is expressed in OASCs contrary to ABASCs (an average of 8.3% compared to 0.7% of expression in ABASCs) (Figures 1(a)–1(c)). OASCs showed multipotency ability by their capacity to differentiate adipocytes, as evidenced by high staining Oil Red O and in a lesser extent into osteocytes as shown by Alizarin Red staining (Figures 1(d) and 1(e)).

### 2.2. OASCs-CM Inhibits RPE Cell Death under Oxidative Stress

**Figure 2:**
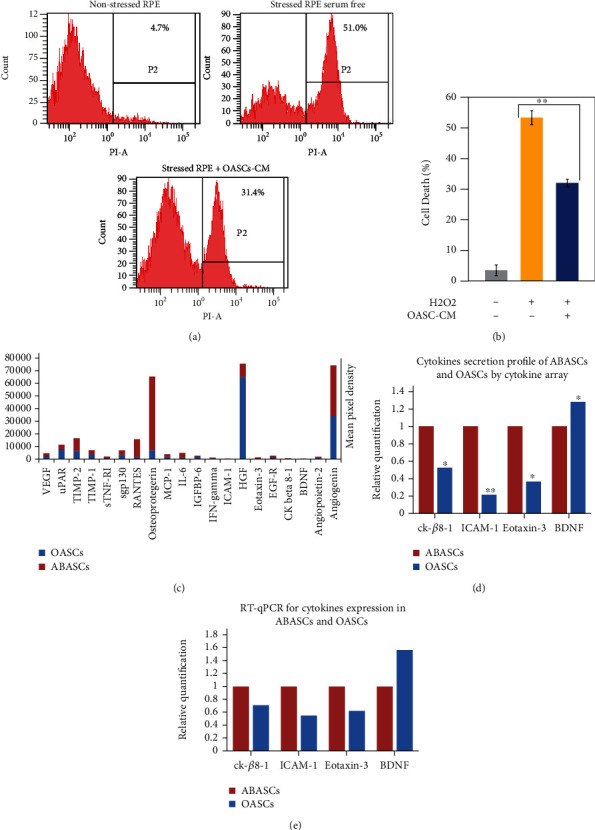
Paracrine activity of OASCs and ABASCs. (a, b) OASCs rescue RPE cells under oxidative stress. RPE cells were incubated with CM or with non-CM for 48 hours, followed by exposure to H2O2 (1 mM, 7 h). Cells were harvested, and cell death rate was analyzed by PI staining followed by flow cytometer analysis. Data are represented as mean ± SD. (c) Medium containing secreted protein of OASCs and ABASCs was examined using RayBio® Human Cytokine Antibody Array G10. Cytokine array membranes used to analyze the secretion of 20 cytokines by OASCs and ABASCs. (d) Summary of significant released proteins elevated in OASCs compared to ABASCs. (e) Gene expression of these proteins in OASCs compared to ABASCs. CM: condition medium.

We assessed the protective role of OASC–conditioned medium (CM) on RPE cells previously exposed to H_2_O_2_ as described for ABASCs-CM [10]. RPE cells exposed to CM prior to H_2_O_2_ treatment exhibited a significant decrease in cell death (32.0 ± 4.1% cell death) compared to non-CM (53.4 ± 4.2% cell death), as evident by FACS analysis for propidium iodide staining (40.0% cell death reduction, *p* < 0.01) (Figures 2(a) and 2(b)). This result highlights the effect of OASC-conditioned medium on oxidation-induced RPE cell death.

### 2.3. Cytokine Secretion Profile of OASCs and ABASCs

Mesenchymal stem cells exert their effect on their microenvironment by paracrine release [9, 10]. In order to examine the cytokine secretion profile of OASCs and to compare that of ABASCs, we used the RayBio® Human Cytokine Antibody Array G10. The most abundant proteins secreted in all samples by OASCs and ABASCs were angiogenin, HGF, and osteoprotegerin, previously described for their inhibitory effects on oxidative stress [21] and apoptosis [22, 23] (Figure 2(c)). Some cytokines were significantly less released by OASCs (Ck-*β*8-1, ICAM-1, eotaxin-3) than ABASCs while the neuroprotective growth factor BDNF was significantly more secreted into OASCs-CM (Figure 2(d)). qRT-PCR was performed to further validate these results on mRNA levels of selected cytokines. To summarize, OASCs exhibited a lower expression of the proinflammatory and proangiogenic cytokines ck-*β*8-1, ICAM-1, and eotaxin-3 than ABASCs (by, respectively, 1.29-, 1.45-, 1.38-fold). Moreover, a higher expression of the neuroprotective factor BDNF was detected in OASCs by 1.57-fold than ABASCs (Figure 2(e)).

### 2.4. Differentiation of OASCs to RPE

#### 2.4.1. Prestaining for Lineage Marker of OASCs/ABASCs and pRPE

**Figure 3:**
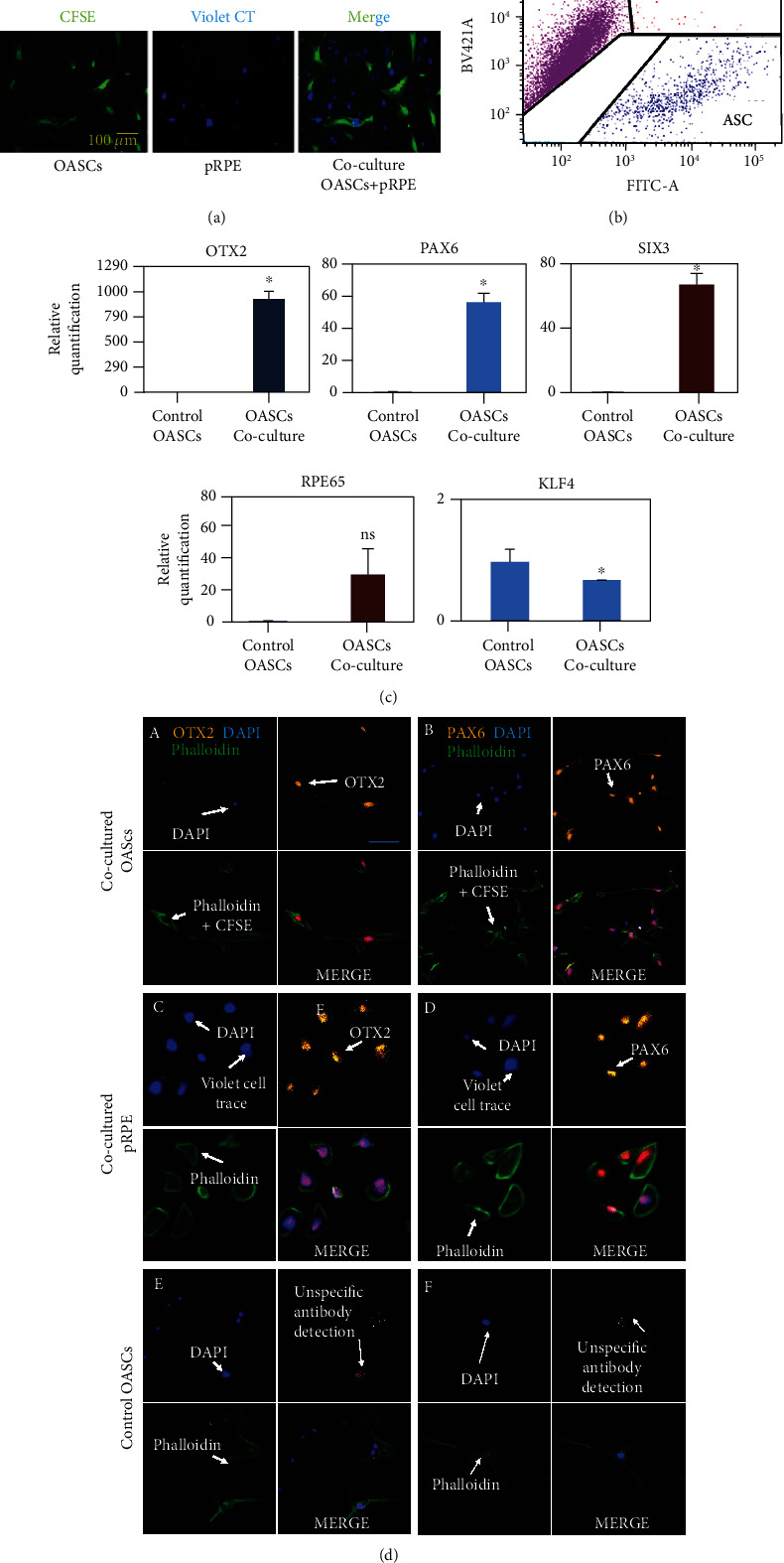
Differentiation of OASCs to RPE like cells. (a, b) Coculture of p-RPE cells and OASCs. (a) Prestaining of cells: OASCs were marked with CSFE (green color), and RPE mature cells were marked with Violet Cell Trace (blue color). (b) FACS sorter: three distinct populations are defined after coculture: RPE cells, OASCs and OASCs, and RPE cells which were fused together. Autofluorescent cells were excluded according to negative and positive controls of labeled cells. (c) Evidence of differentiation of OASCs to RPE like cells by RT-qPCR. Results are presented as mean increase ± SD relative to sample plot. After coculture with RPE cells, OASCs exhibited an increase in transcription of early and late RPE markers. (d) Evidence of differentiation potential of OASCs by immunofluorescence. (A) Sorted OASCs derived RPE-like cells labeled with CFSE showing nuclear localized OTX2 expression. (B) Sorted OASCs derived RPE-like cells showing nuclear localized PAX6 expression (C) Positive control of sorted RPE cells in Violet Cell Trace showing nuclear localized OTX2 expression. (D) Positive control of sorted RPE cells in Violet Cell Trace showing nuclear localized PAX6 expression. (E, F) Negative control of OASCs that did not show any OTX2 expression or PAX6 expression. CT: cell trace. Scale bar: 100 *μ*m. *n* = 3.

**Figure 4:**
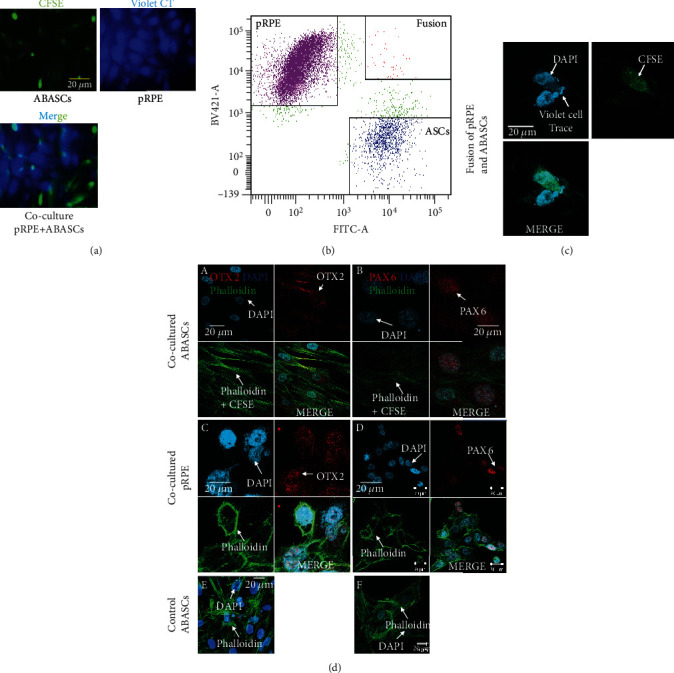
Differentiation potential of ABASCs to retinal precursors cells. (a, b) Coculture of p-RPE and ABASCs. (a) Prestaining of cells: ABASCs were marked with CSFE (green color), and RPE mature cells were marked with Violet Cell Trace (blue color). (b) FACS sorter: three distinct populations are defined after coculture: RPE, ABASCs, RPE and ABASCs which were fused together. (c) Fusion of cells as shown by one cell expressing both Violet Cell Trace (RPE tracer) and CFSE (OASC tracer). (d) Evidence of differentiation potential of ABASCs to retinal precursors cells by immunofluorescence. (A) Sorted ABASCs expressing cytoplasmic OTX2. (B) Sorted ABASCs in CFSE showing nuclear localized PAX6 expression. (C, D) Positive control of sorted RPE cells in Violet Cell Trace showing nuclear localized OTX2 expression and nuclear localized PAX6 expression (E, F) negative controls of undifferentiated ABSCs does not show any OTX2 expression and PAX6 expression. CT: cell trace. *n* = 3.

OASCs/ABASCs and pRPE were prelabeled with CFSE and CellTrace Violet, respectively (Figures 3(a) and 4(a)) and were cell sorted by FACS sorter after a week in coculture. Three well-defined homogenous populations were detected: CFSE+ cells (OASCs/ABASCs), violet cell trace+ (pRPE cells), and a rare population of double-positive CFSE+/Violet CellTrace+ (OASCs/ABASC-pRPE-fused cells) (Figures 3(b) and 4(b)). Segregated cells were collected and analyzed for RPE markers by qRT-PCR and immunostaining.

#### 2.4.2. Increase of Early RPE Markers in OASCs and ABASCs following a Differentiation Study

Relative mRNA expression of cocultured OASCs compared to non cocultured OASCs control is presented in Figure 3(c). OASCs upregulated early neural marker OTX2 (929.7 ± 76.2, *p* < 0.05); all three are markers of early eye field differentitaion. OASCs also upregulated RPE65 (30.5 ± 14.8, *p* = 0.076), a late differentitaion marker of RPE (24) PAX6 (57.6 ± 5.6, *p* < 0.05), and SIX3 (338.2 ± 33.0, *p* < 0.05), and all three are markers of early eye field differentiation and late RPE marker RPE65 [24] (30.5 ± 14.8, *p* = 0.076). We noticed downregulation of the pluripotency marker KLF4 which may suggest the decrease in stemness and commitment to a certain lineage [24] (0.68 ± 0.02, *p* < 0.05) (Figure 3(c)). These results may imply to increase of early RPE markers in OASCs, after coculture with primary RPE cells. To further validate the qPCR results at the protein level, we examined by immunocytochemistry the phenotype of coculture cell-sorted OASCs (Figure 3(d)). The immunostaining analysis tend to confirm the capacity of differentiation of OASCs, as shown by the nuclear expression of PAX6 and OTX2 in OASCs (Figure 3(d)(A)–(D)) when compared to noncocultured OASC control (Figure 3(d)(E, F)).

To better understand whether or not OASCs have an advantage in differentiation to RPE, we compared the differentiation capacity of ABASCs to that of OASCs under the same differentiation protocol. To this end, ABASCs were cocultured with p-RPE cells as previously described in methods. After their coculture with p-RPE cells, ABASCs were able to express the eye-field and neural markers PAX6 and OTX2 exhibiting a potential of early differentiation to retinal precursors. It was observed that PAX6 was expressed in sorted ABASCs in the nuclear region (Figure 4(d)(B)) as observed in sorted OASCs (Figure 3(d)(B)); however, the expression of OTX2 was cytoplasmic in ABASCs (Figure 4(d)(A)) unlike nuclear expression in RPE cells [25] (Figure 3(d)(C, D)) and in differentiated OASCs (Figure 3(d)(C)), a fact that may imply on different differentiation capacity of this population of cells [25]. We also noticed the presence of RPE-ABASC fused cells (Figure 4(c)).

### 2.5. Retinal Lineage Specific Therapeutic Effect of ABASCs and OASCs

To determine the potential of ABASCs and of OASCs in mending retinal injury caused by oxidative stress, we injected those cells to the subretinal space in a sodium iodate mice model, known to describe a retinal degeneration [26].

#### 2.5.1. Cell Location following ASC Transplantation

Transplanted ASCs were detected in the subretinal space at day 0 by hematoxylin and eosin staining (H&E), thus confirming accuracy of transplantation to the desired location (supplementary Figure 1).

#### 2.5.2. Effect of ABASC Transplantation on Photoreceptors

ONL thickness and rhodopsin intensity were previously shown to be reduced after a week of 50 mg/kg sodium iodate [10] while INL thickness was reduced after 3 weeks of NaIO3 (sodium iodate) incubation [27] in untreated NaIO3 model.

**Figure 5:**
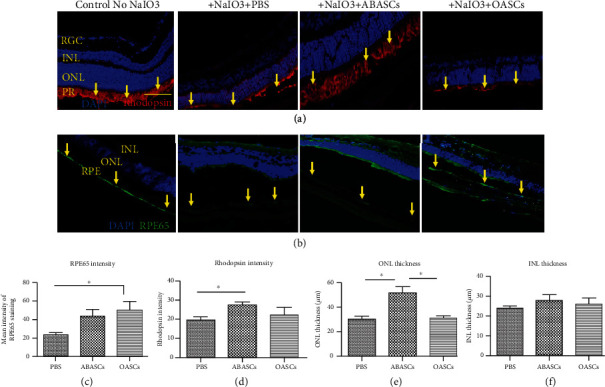
Retinal layers 21 days post transplantation of ABASCs and OASCs in the NaIO3 mice model. (a) Immunohistochemistry pictures of mice retinas for ONL quantification and rhodopsin intensity by DAPI and rhodopsin staining in ABASCs and OASCs transplanted mice versus controls. Yellow arrows indicate the PR layer. (b) Immunohistochemistry pictures of the mice RPE layer after RPE65 and DAPI staining in ABASC and OASC transplanted mice versus controls. Yellow arrows indicate the RPE layer. (c) Graphic summary of RPE65 intensity evident by RPE65. (d) Graphic summary of rhodopsin intensity evident by rhodopsin staining. (e) Graphic summary of ONL thickness evident by DAPI staining. (f) Graphic summary of INL thickness evident by DAPI staining. ONL: outer nuclear layer; INL: inner nuclear layer; RGC: retinal ganglion layer; PR: photoreceptor; RPE: retinal pigment epithelium. Bar = 100 *μ*m. For each group of mice, *n* = 5. Data are represented as mean ± SEM.

ONL thickness as well as rhodopsin intensity was significantly higher in ABASC-treated mice, when compared to the PBS-treated group. Also, ONL thickness was higher in ABASC-treated mice, when compared to the OASC-treated group. No significant difference was found with ONL thickness or rhodopsin intensity in OASC-treated mice (Figures 5(a), 5(d), and 5(e)). ABASC and OASC transplantations did not affect the inner nuclear layer (INL) (Figure 5(f)).

#### 2.5.3. Effect of OASC Transplantation on RPE Layer

RPE65 is often used as a marker of RPE functionality [10, 27]. RPE65 was shown to be reduced after a week of 50 mg/kg NaIO3 [10, 28]. Eyes treated with OASCs showed significantly higher levels of RPE65 staining compared to PBS-treated eyes, exhibiting a protective effect of OASCs from NaIO3-induced damage. No significant difference was observed between ABASC-treated mice to OASCs and PBS-treated mice (Figures 5(b) and 5(c)).

#### 2.5.4. ABASC Transplantation Induced Infiltration of Iba1+ Cells

**Figure 6:**
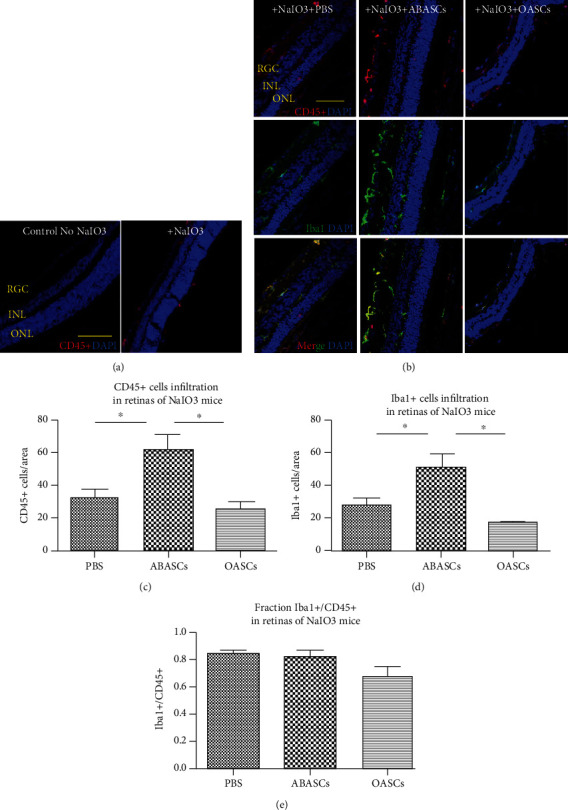
Immune response 21 days post transplantation of ABASCs and OASCs in the NaIO3 mice model. (a) Immunohistochemistry pictures of CD45+ staining in NaIO3-treated mice compared to nontreated mice revealed the infiltration of CD45+ cells in the retinas of NaIO3-treated mice compared to controls. (b) Immunohistochemistry pictures of CD45-Iba1 costaining. (b, c) Quantification of the number of CD45+ cells revealed a higher infiltration of leucocytes in ABASC-treated mice compared to PBS treated mice (*p* ≤ 0.05). (b)–(d) Quantification of the number of Iba1+ cells revealed a higher infiltration of Iba1+ cells in ABASC-treated mice compared to OASC-treated mice and to PBS-treated mice (*p* ≤ 0.05). (b)–(e) Most of CD45+ cells expressed Iba1 in all treated groups. ONL: outer nuclear layer; INL: inner nuclear layer; RGC: retinal ganglion layer. Bar = 50 *μ*m. For each group of mice, *n* = 5. Data are represented as mean ± SEM.

We demonstrated basal CD45+ cell infiltration in NaIO3 mice prior to transplantation of OASCs and ABASCs (Figure 6(a)), as was previously shown [29]. 21 days after transplantation of ABASCs to the subretinal space, we observed an increase of CD45+ cell infiltration (Figures 6(b) and 6(c)) combined with an increase of Iba1+ cells in the retinas of ABASC-treated mice (Figures 6(b) and 6(d)). Iba1+ cells comprised the majority of infiltrating cells in both ABASC- and OASC-treated mice compared to controls (Figures 6(b) and 6(e)). There was no significant difference in infiltration of CD45+ cells and Iba1+ cells in OASC-treated mice compared to PBS-treated mice.

## 3. Discussion

In this study, we characterized the phenotype of OASCs, we demonstrated the protective effect of OASCs on RPE cell death in the setting of oxidative stress, and defined their cytokine secretion profile compared to that of ABASCs. Next, we showed that OASCs were able to express early RPE markers, while ABASCs expressed less specific retinal markers, after coculture with primary RPE cells. Finally, we demonstrated a lineage specific therapeutic effect of OASC and ABASC transplantation in a mouse model of retinal degeneration.

### 3.1. Paracrine Activity of OASCs and ABASCs

Treatment of RPE with conditioned medium of OASCs resulted in salvage of RPE from cell death induced by oxidative stress. These data are in line with a previous study conducted by our group that demonstrated a protective effect of ABASCs on RPE cell death [10]. Also, the contribution of cell fusion may supply this protective effect. In our differentiation study, we found that small percentage of OASCs and ABASCs undergo cell fusion with mature RPE. Those hybrid cells may have an advantage on activating antiapoptotic pathways [30]. OASC and ABASC protective effect might be allowed by high secretion of some cytokines that has been demonstrated in this study. We found in OASCs' and ABASCs' conditioned medium high levels of osteoprotegerin, a cytokine known in the protection from oxidative stress [21]. Also, we showed that OASCs and ABASCs secrete high levels of angiogenin and HGF whose antiapoptotic roles have been elucidated [22, 23]. Moreover, HGF is known for its involvement in tissue regeneration [28] which may explain the beneficial effect seen in vivo in this study.

### 3.2. Variability in Retinal Marker Expression between OASCs and ABASCs following a Differentiation Study

We demonstrate an increase of the early RPE marker expression in OASCs and a raise in less specific retinal lineage marker expression in ABASCs. Immunocytochemistry studies revealed specific nuclear staining to PAX6 and OTX2 in OASCs, implying to obvious lineage direction of OASCs towards RPE [31]. Interestingly, we found that ABASCs had a cytoplasmic expression of OTX2 in contrary to the nuclear expression of OTX2 in RPE cells [25] and in sorted OASCs. A cytoplasmic expression of OTX2 was previously correlated with immature rods photoreceptors [25], as well as the expression of SIX3 and PAX6 [32], a fact that may imply to a potential of ABASCs to follow a path towards precursors of photoreceptors lineage.

Moreover, it was previously shown that stem cells could memorize epigenetic marks from their origin [33], and it might improve RPE differentiation tendency of OASCs [34]. The mutual embryonic ectodermal origin of OASCs and RPE compared to mesodermal origin of ABASCs [35] may explain the increase in RPE markers seen in OASCs, exhibited by the nuclear expression of OTX2 when compared to the cytoplasmic expression of OTX2 by ABASCs.

To note, we found a population of cell fusion of RPE with ABASCs and with OASCs. Although cell fusion was a rare phenomenon of less than 0.7% of cases, it may have some implications. First, this may explain why we found increase in RPE65 transcript in the qRT-PCR study only a week after the differentiation study, since RPE65 is known as a mature RPE markers and usually upregulated later during the course of differentiation [24]. Secondly, the phenomenon of cell fusion may be also an opportunity for enhanced regenerative effect, as previous studies have shown that stem cell fusion with mature differentiate cells promotes the increase of plasticity of mesenchymal stem cells leading and promoting their differentiation [36]. Also, it helps the reprogramming of somatic nuclei through epigenetic changes in a matter that form a pluripotent hybrid cell [37], thus providing another regenerative potential by the reprograming of mature cells to activate selective survival pathways [31].

### 3.3. Long-Term of Subretinal Transplantation of OASCs and ABASCs Leads to Restoration of Different Retinal Layers

As opposed to a recent report by Kuriyan and colleagues, in which transplantation of nonselected mixture of whole adipose tissue cells was injected to the vitreous of AMD patients and caused devastating results, the population of stem cells transplanted in this study was carefully segregated from the whole adipose tissue by a two-step procedure. First, mesenchymal stem cells were isolated from the adipose tissue by collagenase digestion. Second, cells were seeded on tissue culture plate and further purified with specific medium which allowed the selection of purified MSCs. MSC phenotype was then verified by FACS analysis, as mentioned in methods. Moreover, purified MSCs were injected to the subretinal space rather than to the vitreous cavity to reduce the risk of retinal detachment as observed by Kuriyan and colleagues [38].

It is known that NaIO3 injection causes degeneration of ONL and INL [27].We previously reported that subretinal transplantation of ABASCs in NaIO3 mice significantly increased ONL thickness, rhodopsin intensity, and salvaged RPE layer acutely, after one week [10]. In this study, we demonstrate a longer term effect of ABASC transplantation, which showed increase of ONL thickness and rhodopsin intensity after three weeks from NaIO3 administration. However, ABASC transplantation did not result in significant increase of RPE layer after 3 weeks. This may be explained by the destructive effect of NaIO3 on the RPE that is known to be increased with time [39], combined with the fact that ABASCs were shown here to have a more limited ability to increase specific RPE markers than OASCs. Also, OASC transplantation did not affect ONL thickness neither rhodopsin intensity in NaIO3 mice, that might be explained by the differences noticed in immune cell infiltration in retinal layers after ABASC and OASC treatment. ABASC and OASC injections did not result in restoration of the INL layer, whose destruction might be irreversible after a long incubation with high concentration of sodium iodate as shown by Moriguchi et al. [39]. We detected injected ASC at time 0 post transplantation in the subretinal space by H&E staining. However, ASCs were not localized at day 21 post transplantation, neither by H&E staining nor by staining with the human specific markers Ku80 and STEM121 [40]. Nevertheless, the therapeutic effect of each population of transplanted cells was obvious in this study. The explanation to this phenomenon may reside in the strong paracrine effect of ASCs that may have led to a cascade of events resulting in preservation of the RPE layers and photoreceptors layers. Transplanted cells' viability, retention, and engraftment in the tissue have been already described as one of the main issues in stem cell therapy [41] and specifically in retinal transplantation of stem cells [42]. Similar observations of paracrine effect post transplantation were previously described after intra-articular injection of MSCs [43], and many studies are focusing on MSC secretome to improve MSC therapeutic effect in the injured tissue [44, 45]. A completion study to the current investigation will be transplantation of ASCs embedded in a bioscaffold to better improve the survival and retention of the cells in the subretinal space for a longer period of incubation.

### 3.4. ABASC Transplantation Triggers Immune Cell Infiltration in SI Mice

We observed higher CD45+/Iba1+ cell infiltration in retinal layers after ABASC treatment compared to the PBS group and to OASC group. The implication of microglial cells is correlated with neuroinflammation which triggers the increase of the CD45 expression [46]. Microglial Iba1+ cells have a significant impact on neurons viability and function in either beneficial or detrimental effect, depends on the circumstances [47, 48]. Interestingly, the infiltration of microglial cells is known to be involved in photoreceptor cell death protection [48, 49]. Thus, Iba1+ cell infiltration in photoreceptor layers of ABASC-treated mice might be responsible for the regeneration of photoreceptors layer demonstrated in this study. Interestingly, we showed that ABASCs highly secrete ICAM1, eotaxin 3, and Ck*β*-8 that have been previously described to activate microglial infiltration into the brain [50–53]. This secretion may be directly correlated with the higher infiltration of Iba1+ cells in ABASC-treated mice. The absence of Iba1+ cell infiltration increase in mice treated with OASCs may come in line with the absence of photoreceptor protection in this group. Consequently, it might be interesting to study the effect of ABASCs after Iba1+ cell depletion in the sodium iodate mice model.

We demonstrated preservation of the RPE layer in mice treated with OASCs compared to mice treated with ABASCs or controls of PBS. RPE is one of the contributors to the integrity of the outer blood retinal barrier (oBRB). RPE also exhibits immunosuppressive properties by paracrinely preventing the traffic of cells and proteins from the blood to the retinal region [54]. Therefore, preservation of the RPE layer in mice treated with OASCs may explain the lower Iba1+ cell infiltration exhibited in this group. In summary, OASCs exhibited a specific ability to increase the expression of early RPE markers and a protective effect on RPE in vitro and in vivo. When comparing the therapeutic potential of OASCs versus ABASCs, the criteria of abundance and easy accessibility [14] as seen for ABASCs are considered as a necessity for future development of clinical applications. This, combined with a strong in vivo neuroprotective effect, and the increase in retinal lineage markers by ABASCs demonstrated in this study, implies to a broader therapeutic effect and a possible advantage related to ABASCs as a therapeutic tool. All these data imply for spatial effect of either stem cell lineage, OASCs, or ABASCs, on retinal regeneration, and distinguish the potential of each population in future development of cell therapy for retinal diseases.

## 4. Materials and Methods

The purpose of the study and the procedures used were presented to all of the subjects, and a signed informed consent was obtained from each. This study was approved by the ethics committee for clinical trials of Tel Aviv Sourasky Medical Center and was conducting according to the Declaration of Helsinki.

### 4.1. Isolation, Characterization, and Culture of ASCs

Subcutaneous abdominal human adipose tissue was harvested from 4 healthy patients with a mean age of 51 ± 4.2 who had abdominoplasty. Central and medial orbital fat was harvested from 7 patients with a mean age of 72.66 ± 13.57, who underwent routine blepharoplasty. No severe ophthalmopathy, metabolic disease, systemic complications, or recent exposure to chemotherapy or to immunosuppressive agents were reported for these patients. OASCs and ABASCs were isolated according to a protocol previously described [14]. Briefly, the abdominal adipose tissue was extracted by liposuction while orbital fat was extracted during blepheroplasty. The fat tissue was then washed with phosphate-buffered saline (PBS) and digested with collagenase I (0.075% collagenase type I, Sigma-Aldrich, Taufkirchen, Germany) for a 16-hour digestion at 37°C and neutralized with Dulbecco's Modified Eagle Medium (DMEM) containing 10% foetal bovine serum (FBS). Digested fat was centrifuged at 400 g (RCF) for 15 minutes, and the pellet of cells was resuspended in ADSC medium after passage through a 100 *μ*m mesh filter (Cat. no. 542000, EASYstrainer, Greiner Bio-One) to remove all the debris. Cells were then seeded on tissue culture plates. Cell culture medium was replaced twice weekly. All experiments were performed at passage three.

### 4.2. Characterization of OASCs for MSC Markers by FACS Analysis

Characterization of cultured OASCs was performed at passages 3-4 as follows: after reaching 100% confluence, cells were trypsinized and collected in FACS tubes in aliquots (1 × 10^5^ cells/tube). Cells were stained with fluorescein isothiocyanate (FITC) and phycoerythrin- (PE-) conjugated monoclonal antibodies against human CD34 (Dako), CD45 (Dako), CD90 (Dako), CD105 (eBioscience), and CD73 (BDPharmingen). To exclude dead cells, the samples were stained with ViViD (violet viability dye, Molecular Probes, Invitrogen, Eugene, OR, USA), according to the manufacturers protocols. Cells were then analyzed by FACS Canto II flow cytometer (BD Biosciences). All the fluorochrome-conjugated antibodies were used as isotype controls.

### 4.3. Multipotency of OASCs by Differentiation to Osteocytes and Adipocytes

OASCs at passage 3 were studied to determine their ability to differentiate into osteocytes and adipocytes. 1 × 10^4^ cells were plated in a 24-well plate and incubated with their respective differentiation media after 100% of confluency (adipocyte: 10% FBS, 1 *μ*M dexamethasone, 0.5 mM 3-isobutyl-1-methylxanthine, 10 *μ*g/mL insulin, and 100 *μ*M indomethacin in high glucose- (HG-)DMEM, osteocyte: Stem pro® osteocyte differentiation basal medium (Gibco)). The media was changed twice a week until osteocyte and adipocyte differentiation, after 2 or 3 weeks, respectively. Differentiation to adipocytes was assessed using an Oil Red O stain as an indicator of intracellular lipid accumulation. The cells were fixed for 20 min at room temperature in 4% paraformaldehyde. Cells were incubated in 0.5% (wt/vol) Oil Red O reagent in 100% isopropanol (Sigma) for 10 min at room temperature. Bone differentiation was followed by Alizarin Red Staining (Sigma) after fixation with 4% paraformaldehyde. Images of stained cells with both Oil Red O and Alizarin red were taken by light microscopy.

### 4.4. Primary RPE (pRPE) Culture and Preparation of ASC-Conditioned Medium

5.5 × 10^5^ cells of human pRPE cells (Lonza) were plated in 100 mm culture dishes (Falcon) in retinal epithelial cell growth medium (RtEGM) Bulletkit (Lonza) and incubated at 37°C in a humidified 5% CO_2_ atmosphere. The medium was replaced twice weekly, and cells were passaged with 0.25% trypsin/0.05% EDTA (Biological Industries, Israel) upon reaching 90%confluence. Experiments were performed at passage three. OASCs and ABASCs (1.0 × 10^6^ cells) at passage 3 were plated on a 100 mm dish (Falcon) and cultured in ASCs BulletKit™ Medium (Lonza). At 100% confluence, ASCs were washed with PBS and cultured with ASC serum free medium (Lonza) for 48 h before isolation of their respective medium, containing many released growth factors and cytokines. Both OASC-conditioned medium (OASC-CM) and ABASC-conditioned medium (ABASC-CM) were maintained at -80°C for further analysis using protein Array (RayBiotec).

### 4.5. Rescue Study

#### 4.5.1. RPE Cells Were Preincubated with OASC-CM Followed by Treatment with H2O2

1 × 10^4^ RPE cells were seeded in a 6-well plate (Falcon), in 1 mL of RtEGM containing 2% FBS (Lonza). The medium was changed to free-FBS RtEGM and renewed every two days until treatments. After reaching approximately 90% confluence, RPE cells were preincubated for 48 hours with either OASCs conditioned medium or with nonconditioned ADSC serum free medium (non-CM) as control. RPE cells were then washed with PBS followed by exposure to 1.5 mM H_2_O_2_ (Cat. no. 216763, Sigma) or without H_2_O_2_ exposure as control. After 7 hours, RPE cell death was monitored by propidium iodide (PI) using FACS analysis.

#### 4.5.2. Propidium Iodide Staining and Flow Cytometry Analysis

Following rescue studies as described above, RPE cells at passage three were harvested with 0.25% trypsin/0.05% EDTA (Biological Industries). Cells (3 × 10^5^) were collected by centrifugation at 500 g for 5 min, washed twice with PBS, and resuspended in 400 *μ*l of PBS to which 1 *μ*l of propidium iodide (1 mg/mL, Sigma) was added immediately before flow cytometry measurements. At least 10,000 events were collected and labeled, and fluorescent cells were detected by BD FACS CantoTM II cytometer (BD Pharmingen, USA). Analysis of cell death distribution was conducted by FCS Express 4 software (De Novo Software, Canada).

#### 4.5.3. Human Cytokine Antibody Array

Cytokine secretion of OASCs was studied by cytokine antibody array on collected culture medium and compared to that of ABASCs. Conditioned medium was prepared as described above; briefly, ASCs' medium was changed to serum-free medium, following 48 hours of incubation, and the medium was collected and centrifuged at 4500 rpm for 5 min and supernatants stored at −80°C until they were assayed. Levels of medium released cytokines and growth factors were measured by RayBio® Human Cytokine Antibody Array G10 (Thermo-Fisher) according to manufacturer's protocol.

#### 4.5.4. Differentiation of OASCs to RPE

We evaluated the differentiation potential of OASCs to RPE using a coculture system [55]. First, each cell line was marked with a different fluorescent dye in order to lineage-trace differentiated cells following coculture. OASCs and p-RPE were, respectively, labeled, with green fluorescent dye using carboxyfluorescein succinimidyl ester (CFSE) from CellTrace™ CFSE Cell Proliferation Kit (Invitrogen) and violet fluorescent dye using CellTrace™ Violet Cell Proliferation Kits (Invitrogen) according to the manufacturers' instructions. Then, OASC (1 × 10^6^) cells were cocultured with primary human RPE (1 × 10^6^) cells for one week in a mixture medium composed by 50% of Dulbecco's Modified Eagle Medium (DMEM) containing 10% foetal bovine serum (FBS) and 50% of free-FBS RtEGM and incubated at 37°C in a humidified 5% CO_2_ atmosphere. Half of the medium was changed two times a week. All the cells were harvested and analyzed with a BD FACSAria Fusion Cell Sorter instrument (BD Biosciences) according to their respective marker. Results were compared to a negative control-OASCs noncocultured with RPE and a positive control-mature primary RPE cells labeled with CellTrace™ Violet (also called Violet Cell Trace).

#### 4.5.5. Characterization of OASCs and ABASCs Cell Sorted after Coculture

After cell sorting of the cells, we identified OASC-derived RPE cells, and we characterized the level of differentiation by qRT-PCR using primers (Table S1) and immunocytochemistry using, respectively, monoclonal/polyclonal antibodies anti-PAX6/anti-OTX1/2 (1/100, Abcam, Cambridge, England), and a goat anti-rabbit Alexa Fluor 594-conjugated crossadsorbed secondary antibody (1/1000, Invitrogen).

#### 4.5.6. Gene Expression Quantification

The gene expression profile of OTX2, PAX6, SIX3, RPE65, and KLF4 in OASCs after costaining with primary human RPE was quantified using qRT-PCR in order to detect differentiation markers. Results were compared to a negative control-OASCs which were non c-cultured with RPE. We also quantified the expression of Ck-*β*8, ICAM1, eotaxin-3, and BDNF in OASCs and ABASCs to confirm the cytokine antibody array. Total RNA was extracted from OASC and ABASC cell cultures using High Pure RNA Isolation Kit (Roche) or TRI Reagent (Sigma-Aldrich) according to the manufacturers' instructions. Total RNA concentration was reverse transcribed using qScript cDNA Synthesis Kit (Quantabio) or Verso cDNA synthesis kit (Thermo-Scientific), and the mRNA expression levels of these genes were measured by RT-PCR (StepOnePlus–Applied Biosystems), using SYBR® Green qPCR Mastermix (Qiagen). Results were normalized by using the housekeeping gene GUSB. Results were calculated by the *ΔΔ*CT method of relative quantitation. List of primers used is presented in Table S1.

#### 4.5.7. Immunocytochemistry

For detection of retinal precursors markers at the protein level, sorted cells and controls were plated on 13 mm coverslips in a 24 well-plate (Falcon) overnight, respectively, in a mixture medium (50% of DMEM 10% FBS/50% of free-FBS RtEGM) or in DMEM containing 10% of FBS and incubated at 37°C in a humidified atmosphere (respectively, 5 and 8% of CO2). The cells were fixed with 4% paraformaldehyde for 20 minutes, permeabilized with 0.1% Triton™ X-100 for 5 minutes, blocked overnight with PBS diluted 2% BSA, and labeled with rabbit primary antibody anti-OTX1/2 (OTX1 is not expressed in RPE and photoreceptors [56]) or PAX6 for 1 hour at room temperature. Goat anti-Rabbit IgG (H + L) Cross-Adsorbed Secondary Antibody, Alexa Fluor 594 (A-11012), and Alexa Fluor® 488 Phalloidin (Invitrogen) were diluted in phosphate-buffered saline containing 2% BSA for 1 hour at room temperature, for detection of OTX1/2 or PAX6. Nuclei were stained with DAPI in Thermo Scientific™ Shandon™ Immu-Mount™.

#### 4.5.8. Animal Procedure

Wild-type C57BL mice received intraperitoneal (IP) injection of 50 mg/kg of sodium iodate (NaIO3) (Sigma-Aldrich) (*n* ≥ 4). The sodium iodate induces oxidative stress and acute injury to RPE that triggers progressive retinal damage [26]. Twenty-four hours after injection, mice were anesthetized, and a small self-sealing sclerotomy was performed with the tip of a 30-gauge needle. A 36-gauge needle attached to a Hamilton syringe (Hamilton Company, Reno, Nevada, USA) was inserted through sclerotomy into the subretinal space, and an injection of 1.5 *μ*l of PBS containing 4 × 10^4^ cells was performed.

Mice received cyclosporine in drinking water for a week after the transplantation, at a concentration of 210 mg/l. Animal handling and experiments were performed following institutional care guidelines with the approval of the Tel Aviv Sourasky Medical Center Animal Ethics Committee.

#### 4.5.9. Tissue Preparation

Mice were euthanized on day zero and three weeks following subretinal transplantation; the eyes were enucleated and fixed in 4% formaldehyde (Merck, Darmstadt, Germany) overnight. The eyes were washed in PBS and then incubated for cryoprotection with 30% sucrose in PBS overnight at 4°C. Fixed tissue was embedded in OCT Tissue Freezing Medium (Scigen Scientific, Gardena, CA, USA) and frozen on dry ice. Cross-sections (10 *μ*m) were placed on X-tra adhesive slides (Leica Biosystems, Peterborough, UK) and stored at -20°C.

#### 4.5.10. Immunohistochemistry

Frozen slides were stained with hematoxylin and eosin (H&E). For immunofluorescence staining, sections were washed in PBS for 20 minutes and blocked with 5% bovine serum albumin (BSA) (Sigma-Aldrich), 1% normal goat serum (NGS) (Invitrogen, no. 31872), and 0.5% Triton X-100 (Sigma-Aldrich) in PBS for 1 hour at room temperature. The sections were incubated overnight with anti-RPE65 rabbit monoclonal primary antibody (ab231782, 1 : 200), anti-CD45 clone IBL-5/25 Rat monoclonal antibody (EMD Millipore, Merck, Darmstadt, Germany, 1 : 50), Mouse anti-rhodopsin monoclonal antibody (EMD Millipore, Merck, Darmstadt, Germany, 1 : 200), Iba1, and rabbit antibody (Wako, Osaka, Japan, 1 : 1000), in blocking solution at 4°C. The slides were washed three times with PBS-0.5% Triton X-100 (PBST), incubated with Alexa Fluor 488 Goat anti-rabbit, Alexa Fluor 546 Goat-anti-rat, and Alexa Fluor 546 Goat anti-mouse (Invitrogen, 1/200) in PBST for 1 h at room temperature in blocking solution, and washed again three times with PBST. The sections were then incubated with the nuclear dye DAPI (Molecular Probes, Thermo Fisher Scientific) for 10 minutes, washed twice in PBS, and mounted in ImmunoMount (Thermo Scientific, 9990412).

Pictures were carried out with a Zeiss LSM 700 confocal microscope in a blinded manner. Analysis was performed by ImageJ software. RPE65 and rhodopsin intensity and quantification of positive CD45 and Iba1 cells were performed by measuring the fluorescence intensity compared to the image's background. Also, the thickness of ONL and INL was evaluated by measuring length of ONL and INL layers (stained with DAPI) in at least 6 points of the layer. The sections shown were selected at the same distance to the optic disc and in the same retinal quadrant.

### 4.6. Statistical Analysis

Statistical analyses were performed using GraphPad Prism software (version 9.0, GraphPad Software, San Diego, California). The Mann–Whitney test was used to analyze nonparametric data. For the mice results, a Kruskal-Wallis nonparametric test was used to identify differences between the three groups. Statistical significance was accepted for *p* value< 0.05.

## Data Availability

Will be given upon request.

## Supplementary Materials

Supplementary Materials Table S1: primers used in quantitative RT-PCR experiments. Supplementary Figure 1: detection of cells in the subretinal space on the day of ASC transplantation in the NaIO3 mice model.
