# Supplementary material for: Retinal Lineage Therapeutic Specific Effect of Human Orbital and Abdominal Adipose-Derived Mesenchymal Stem Cells
Source: Stem Cells Int. 2021 Oct 19;2021:7022247. doi: 10.1155/2021/7022247 (PMC8548122; doi:10.1155/2021/7022247)
Supplement: Supplementary Materials — Table S1: primers used in quantitative RT-PCR experiments. Supplementary Figure 1: detection of cells in the subretinal space on the day of ASC transplantation in the NaIO3 mice model. [file 7022247.f1.docx]

**SUPPLEMENTARY MATERIAL**

| Sequence (5' to 3') R | Sequence (5' to 3') F | Human Gene |
| --- | --- | --- |
| TAACATCTGCAAGCATAAACGACAA | TAAGCCTAGCAGTAAAGAGACATTGG | OTX2 |
| GGTGCTGGAGCCTGTTCTT | CTACAGGACCCCTACCCCAA | SIX3 |
| CAGGGATCTGGGAAAGCACA | ACCACAGAAGGTTCATCCGC | RPE65 |
| CTGGAGCTCTGTTTGGAAGGA | ACGGCTGCCTTGCCTTCT | PAX6 |
| ATGCTCGGTCGCATTTTTGG | CGAACCCACACAGGTGAGAA | KLF4 |
| GTTTCCCTTCTGGTCATGGA | ATTGGCTGGCGATTCATAAG | BDNF |
| TGGGAGGAAACACCCTCTCC | GACCTGGGT GCGAAGCTATG | Eotaxin-3 |
| GCTGCTACCACAGTGATGATGACAA | CAGTGACCATCTACAGCTTTCCGG | ICAM-1 |
| TCAATTCTTCCTGGTCTTGAT | ATGAAGGTCTCCGTGGCTG | ck-β8-1 |
| ATACGGAGCCCCCTTGTCT | CCTGCGTCCCACCTAGAATC | GUSB |

**Table S1 :** List of human primers used for qRT-PCR in this study.


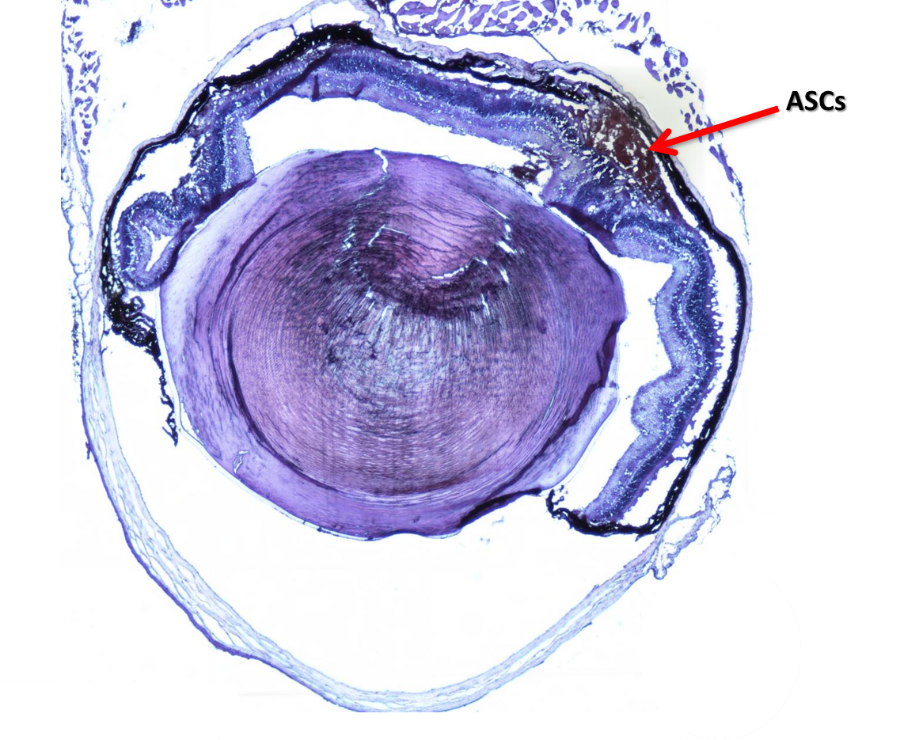


**Supplementary Figure 1. Detection of cells in the subretinal space in NaIO3 mice model on the day of ASCs transplantation.** H&E staining of the retina in NaIO3 mice on the day of ASCs transplantation. ASCs – Adipose derived stem cells, SRS – subretinal space, RPE – Retinal Pigment Epithelium. Magnification X4.
